# Comparison of Efficacy and Willingness to a 5% Gluconolactone‐Based Topical Serum and Intense Pulsed Light in Mild Erythema of Rosacea: A Paired Control Study

**DOI:** 10.1111/jocd.70025

**Published:** 2025-03-20

**Authors:** Yiran Peng, Mengping Mao, Ziyang Huang, Huimin Zhao, Ying Li, Dan Jian

**Affiliations:** ^1^ Department of Dermatology, Xiangya Hospital Central South University Changsha China; ^2^ Hunan Key Laboratory of Aging Biology, Xiangya Hospital Central South University Changsha China; ^3^ National Clinical Research Center for Geriatric Disorders, Xiangya Hospital Central South University Changsha China

**Keywords:** intense pulsed light, mild erythema, paired control, rosacea, topical serum, treatment willingness

## Abstract

**Introduction:**

Mild erythema is a common affliction of rosacea. Many methods including drugs and phototherapy (e.g., Intense Pulse Light, IPL) have been tried to alleviate symptoms. Topical applications for enhancing moisturization have also been thought to be of some benefit. In this study, the improvement of erythema and skin physiology parameters were assessed after a topical serum (5% gluconolactone‐based serum) usage or a single IPL treatment, and the differences in improvement between the two groups were compared. The treatment willingness of the subjects was also performed.

**Methods:**

In this study, 200 subjects with mild erythema were enrolled; 100 of them were treated with topical serum (TS) for 30 consecutive days and the other 100 for a single IPL treatment. Facial images, local skin images, and physiological measurements were collected before and after treatment, to assess erythema (CEA), overall facial condition (VAS), and other skin physiology parameters (e.g., pore, skin tone, and sebum). Based on the questionnaire, the treatment willingness and satisfaction of the subjects were collected.

**Results:**

Improvement on erythema, overall facial condition, and skin physiology parameters were observed in two groups. Both subjective assessments and measurements showed greater efficacy in erythema reduction, overall facial condition improvement, and sebum reduction in the IPL group (*p* < 0.05). Pores and skin tone improvement were comparable in TS and IPL groups. Similarly, subjects with certain erythema improvement in both groups were satisfied with their choice of treatment, in which possible side effects, cost, and duration of the treatment somewhat influence their treatment decisions.

**Conclusions:**

Topical serum that focuses on redness reduction and moisturization and IPL can improve erythema and skin physiological indicators. IPL is more capable of improving the overall facial appearance. By fully understanding the patient's facial condition and treatment influence, medical practitioners determine the appropriate treatment.

**Trial Registration:**

ClinicalTrials.gov identifier: ChiCTR2400087665

## 1 Introduction

Rosacea, as a chronic inflammatory skin disease that affects face, is characterized by persistent erythema, papules pustules, telangiectasia, and flushing. Particularly, persistent erythema disturbs most of the population [1]. According to a cross‐sectional study in China, erythematotelangiectasia is present in up to 47.6% of rosacea [2], which bothers patients both physically and psychologically [3]. Even mild erythema could cause serious psychological effects [4]. It has been reported that a few of mild patients still desired further mitigation of the redness and achieve “clearness” [5, 6].

Recently, more and more phenotype‐based treatments for rosacea have been proposed [7]. Persistent erythema was recommended to be treated with brimonidine tartrate gel (α2‐adrenergic agonist receptor), but it was not applicable to mild erythema due to its side effects. Some patients may be forced to choose physical therapy (intense pulsed light, pulsed dye laser, Nd: YAG lasers, etc.) to alleviate the discomfort of erythema [8]. Intense pulsed light (IPL) is more often chosen because it can improve mild erythema, inhibit sebum secretion, show anti‐inflammatory effect, and lower incidence of purpura and blisters than pulsed fuel lasers [9]. It is also thought that skin barrier function may be impaired in some of rosacea patients and that IPL can further increase trans‐epidermal water loss [10, 11]. Moreover, IPL is expensive, and the cost is outweighed if the erythema worsens [12].

For mild persistent erythema in rosacea, proper skin care plays a pivotal role in maintaining remission, alleviating the symptoms, and improving skin dryness and redness [11]. Patients' facial conditions and their treatment willingness determine their outcome to some extent. This study compared the differences in effectiveness between a topical serum containing PHA (5% gluconolactone and 1% lactobionic acid) and intense pulsed light on various clinical signs and symptoms of rosacea in erythematous rosacea, particularly in terms of reducing erythema. The attitudes of patients toward their treatment options and the underlying factors that may influence their decision‐making process were also explored.

## 2 Methods

This study was performed as a matched retrospective study which was registered in the Chinese Clinical Trial Registry and conducted in accordance with the Declaration of Helsinki. Facial images and skin physiological measurements of patients before and after treatments were retrospectively collected and analyzed.

### 2.1 Study Population

Eligible participants were mild erythematous rosacea patients, whose diagnosis was reconfirmed by at least two professional dermatologists based on the National Rosacea Society Expert Committee [13]. They visited our dermatology clinic and were confirmed by experienced physicians to be eligible for IPL treatment. Some patients opted for IPL treatment, while some refused for personal reasons (treatment costs, fear of treatment, etc.) and chose to use topical serum.

Exclusion criteria were as follows: (1) Incomplete recording of images and skin physiological measurements before and after treatment. (2) Refusal to provide photographs for clinical analyses. All subjects who met the inclusion and exclusion criteria signed an informed consent form.

### 2.2 Materials and Treatment Procedures

Treated patients were routinely photographed by VISIA Complexion Analysis System (Canfield Scientific, Parsippany, NJ, USA) before and at the end of treatment for overall facial images. Partial facial skin images (bilateral zygomatic, bilateral cheek, and forehead) and skin physiology parameters (hydration, elasticity, erythema, pore, sebum, and melanin) were recorded by a skin analysis device (API‐100; Aram Huvis, Gyeonggi‐do, Korea).

Patients were photographed and measured before the first application and 30 days after consecutive use of topical serum (TS), a redness neutralizing serum from NeoStrata Company Inc., Princeton, NJ. IPL‐treated (Broad Band Light (SCITON, USA), 560/590 nm cut‐off filter; energy fluency of 7–9 J/cm^2^; double‐mode; delay time of 20–30 ms) patients were taken pre‐ and post‐treatment on day 30.

### 2.3 Clinical Assessment

Images and physiologic measurements were collected retrospectively. Three independent evaluation investigators (EI) assessed the VISIA images of the participants. The Clinician's Erythema Evaluation Approach (CEA) was used to assess erythema [5] and the severity of the participants' overall facial condition (erythema, pore, skin tone, sebum, etc.). Pre‐ and post‐treatments were assessed using a 10‐point visual analogue scale (VAS) [14, 15], and pores, skin color, and sebum were assessed using a 4‐point VAS (1 to 4) [16]. Skin analysis device shows images and skin physiology parameters in the measured skin. Based on the clinical images and skin physiology parameters pre‐ and post‐treatment, the efficacy within and between groups was evaluated by investigators. The obvious and unobvious improvement percentages of each subjective assessment between two groups were also compared.

For subjects with “obvious improvement” erythema in both groups, representative pictures of the overall condition of both groups with “obvious improvement” and “unobvious improvement” were shown in triplicate at their follow‐up visit and were asked to answer the following questions:(1) “I'm satisfied with the efficacy of the chosen treatment”. (2) “I'd like to choose the other treatment after looking at the comparison images of two groups.” (3) Subjects' reason of the chosen treatment: (a) “I believe in the effect of IPL from friends/social platforms,” (b) “I'm worried about the potential side effects of IPL,” (c) “I don't have much time for hospital treatment,” (d) “I don't want to spend too much money,” (e) “I have no faith in the ability of topical serum to improve facial condition.” They were reported by a 3‐point scale (0: impossible; 1: possible; 2: certain).

### 2.4 Statistical Analysis

The scales for the different measured values of the skin analysis device were unified (Calculated score = measured value/reference value, with a maximum value of 5 (provided by the skin analysis device)). The value used for analysis was the average of five localized facial measurements. Wilcoxon's signed‐rank test was used to analyze the overall facial condition scale (VAS), erythema (CEA), pore, sebum, and skin tone before and after treatment in the topical serum group and IPL group, respectively. The efficacy rate of overall improvement and detailed improvements (erythema, pore, sebum, and skin tone) was examined. Based on paired *t* tests, dermoscopy values were calculated. All hypothesis testing was conducted using two‐sided tests, with *p* = 0.05 level of significance. Data management and analysis were performed using SPSS 25.0 (SPSS Inc., Chicago, IL, USA).

## 3 Results

### 3.1 Participant Demographics

**FIGURE 1:**
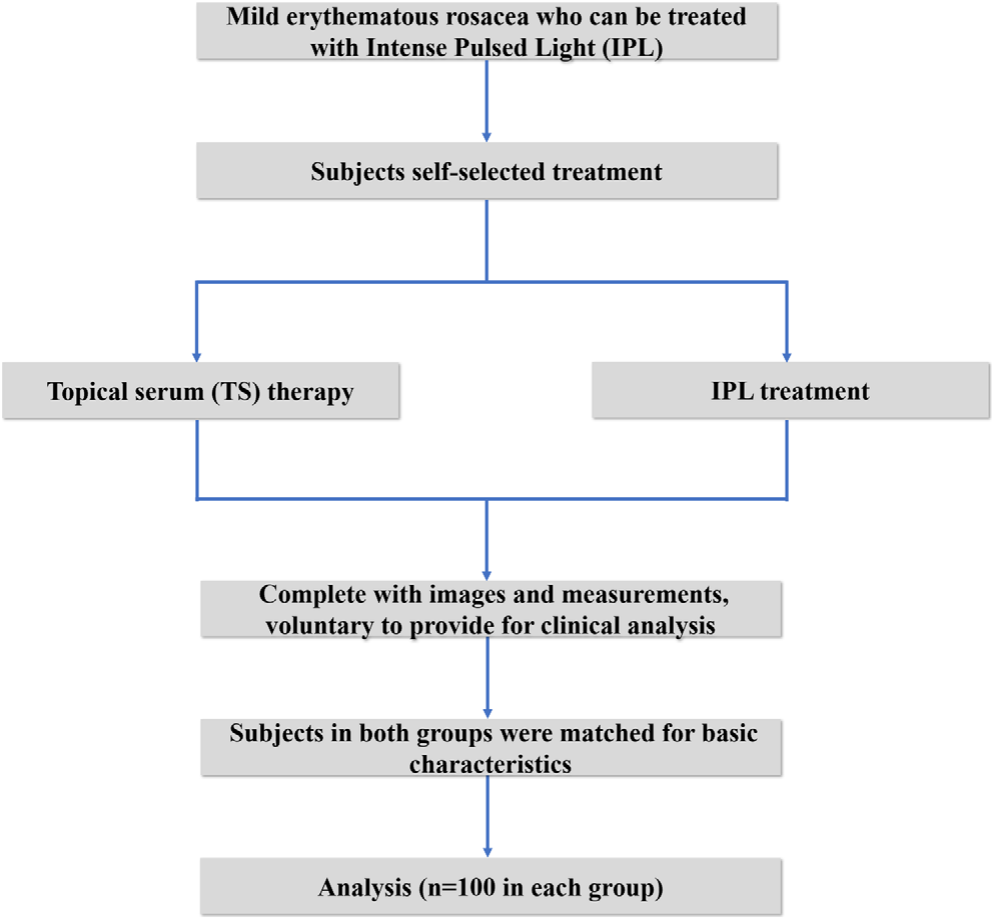
Study flow diagram.

The study design of the current research is illustrated in Figure 1. Patients with similar basic characteristics (age, gender, Baumann skin type, Fitzpatrick skin phototypes, and CEA, *p* > 0.05) treated with topical serum and IPL were selected and divided into TS and IPL groups with 100 individuals each. The population in the trial was predominantly female (*n* = 167, 83.5%), young (from 18 to 32, average: 24.6 ± 3.0), light brown skin (Fitzpatrick skin type IV, 79.0%), rosacea patients. (Table 1).

**TABLE 1:** Demographic and baseline characteristics of trial participants.

	TS (*n* = 100)	IPL (*n* = 100)	Total (*n* = 200)	*p*
Age (years)	24.8 (2.5)	24.6 (3.4)	24.7 (3.0)	0.651
Sex				
Female	85 (85.0)	82 (82.0)	167 (83.5)	0.568
Male	15 (15.0)	18 (18.0)	33 (16.5)	
Skin type (Baumann)				
Combination dry	48 (48.0)	41 (41.0)	89 (44.5)	0.319
Combination oily	52 (52.0)	59 (59.0)	111 (55.5)	
Skin phototypes (Fitzpatrick)				
III	25 (25.0)	17 (17.0)	42 (21.0)	0.165
IV	75 (75.0)	83 (83.0)	158 (79.0)	
CEA (baseline)				
1	27 (27.0)	21 (21.0)	48 (24.0)	0.321
2	73 (73.0)	79 (79.0)	152 (76.0)	
Mean	1.73 (0.45)	1.79 (0.40)	1.76 (0.43)	

*Note:* Data are *n* (%) or mean (SD). 1 = almost clear; slight redness. 2 = mild erythema; definite redness.

Abbreviation: CEA, clinician erythema assessment.

### 3.2 Efficacy

#### 3.2.1 Comparison of Efficacy Within and Between TS and IPL Groups—Investigator Assessment

**FIGURE 2:**
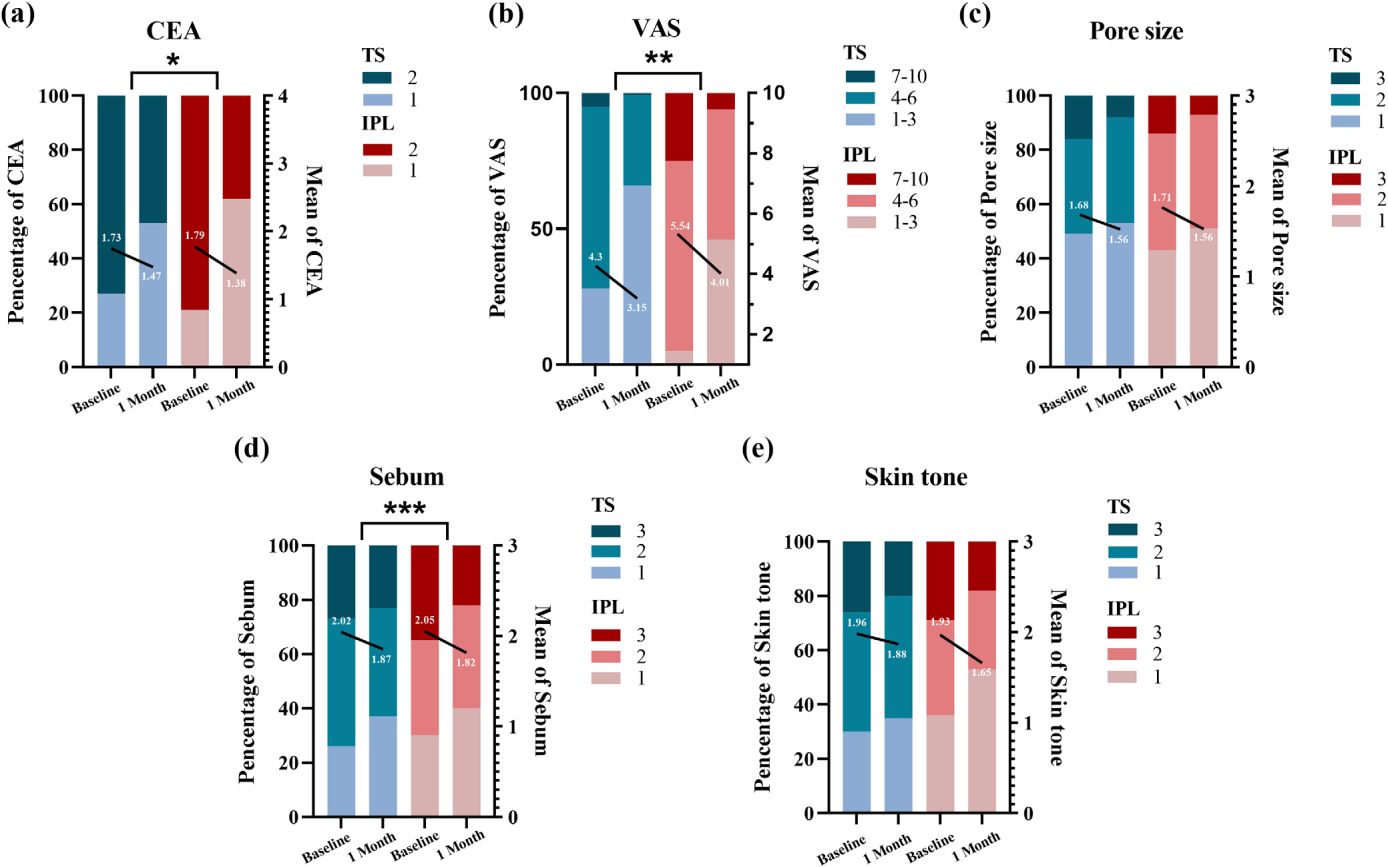
Facial condition subscale scores in the TS‐ and IPL‐treated groups at baseline and 1 month. Percentage (bar) and mean (straight line) changes in indicator scores at baseline and after 1 month in TS and IPL groups. Comparison of the variations (ΔT_a_ = T_Baseline_−T_1 Month_) of each item between the two groups; significance is defined as *p* < 0.05. 0.01 ≤ *p* < 0.05: *; 0.001 ≤ *p* < 0.01: **; *p* < 0.001: ***.

The overall facial condition and corresponding sub‐entries of the two groups at baseline and after 1 month of treatment are shown in Table 2 (Figure 2), with higher scores being more severe. In both groups, the severity of erythema was predominantly mild (CEA = 2), 73% in TS group and 79% in IPL group. Within both groups, there was a statistically significant decrease in CEA, VAS, sebum, pores, and skin color scores before and after treatment (*p* < 0.05). The variations (ΔT_a_ = T_Baseline_−T_1 Month_) of above items’ scores were assessed to compare the efficacy of the two treatments. Statistically significant difference was shown in CEA, VAS, and sebum (*p* < 0.05).

**TABLE 2:** Facial condition subscale scores in the TS‐ and IPL‐treated groups at baseline and 1 month.

Items	TS (*n* = 100)			IPL (*n* = 100)			△T_a_ TS vs. IPL^a^
	Baseline	1 Month	*p*	Baseline	1 Month	*p*	*p*
CEA, *n* (%)							
1	27 (27.0)	53 (53.0)	**< 0.001**	21 (21.0)	62 (62.0)	**< 0.001**	**0.037**
2	73 (73.0)	47 (47.0)		79 (79.0)	38 (38.0)		
Mean (SD)	1.73 (0.45)	1.47 (0.5)		1.79 (0.40)	1.38 (0.49)		
VAS, *n* (%)							
1–3	28 (28.0)	66 (66.0)	**< 0.001**	5 (5.0)	46 (46.0)	**< 0.001**	**0.007**
4–6	67 (67.0)	33 (33.0)		70 (70.0)	48 (48.0)		
7–10	5 (5.0)	1 (1.0)		25 (25.0)	6 (6.0)		
Mean (SD)	4.30 (1.31)	3.15 (1.16)		5.54 (1.25)	4.01 (1.42)		
Pore							
1	49 (49.9)	53 (53.0)	**0.019**	43 (43.0)	51 (50.0)	**0.004**	0.657
2	35 (35.0)	39 (39.0)		43 (43.0)	42 (43.0)		
3	16 (16.0)	8 (8.0)		14 (14.0)	7 (7.0)		
Mean (SD)	1.68 (0.75)	1.56 (0.65)		1.71 (0.70)	1.56 (0.62)		
Sebum							
1	26 (26.0)	37 (37.0)	**0.011**	30 (30.0)	40 (40.0)	**< 0.001**	**< 0.001**
2	47 (47.0)	40 (40.0)		35 (35.0)	38 (38.0)		
3	27 (27.0)	23 (23.0)		35 (35.0)	22 (22.0)		
Mean (SD)	2.02 (0.74)	1.87 (0.77)		2.05 (0.81)	1.82 (0.77)		
Skin tone							
1	30 (30.0)	35 (35.0)	**0.041**	36 (36.0)	53 (53.0)	**< 0.001**	0.105
2	44 (44.0)	45 (45.0)		35 (35.0)	29 (29.0)		
3	27 (27.0)	21 (21.0)		29 (29.0)	18 (18.0)		
Mean (SD)	1.96 (0.76)	1.88 (0.74)		1.93 (0.81)	1.65 (0.77)		

*Note:* Data are *n* (%) or mean (SD). Bold values indicate that the improvement in the corresponding indicators after treatment with the topical serum and IPL were statistically significant.

^a^ △T_a_ TS vs. IPL: compare the variations (ΔT_a_ = T_Baseline_−T_1Month_) of each item between the two groups.

Furthermore, the improvement of each item was categorized as “Obvious” and “Unobvious” to clarify the difference in efficacy between the two groups (Table 3). The TS group was as good as the IPL group in pore reduction (56% vs. 64%, NS) and improving skin tone (57% vs. 65%, NS). “Obvious” proportions of overall improvement, erythema reduction, and sebum reduction were also high in the TS group but better in the IPL group (*p* < 0.05).

**TABLE 3:** Difference in detailed improvements evaluated by investigators at 1‐month post‐treatment between the TS‐ and IPL‐treated groups.

Items	TS (*n* = 100)	IPL (*n* = 100)	*p*
Erythema reduction			
Obvious	56 (56.0)	79 (79.0)	0.001
Unobvious	44 (44.0)	21 (21.0)	
Overall improvement			
Obvious	49 (49.0)	66 (66.0)	0.022
Unobvious	51 (51.0)	34 (34.0)	
Pore size reduction			
Obvious	56 (56.0)	64 (64.0)	**0.248**
Unobvious	44 (44.0)	36 (36.0)	
Sebum reduction			
Obvious	38 (38.0)	53 (53.0)	0.033
Unobvious	62 (62.0)	47 (47.0)	
Skin tone improvement			
Obvious	57 (57.0)	65 (65.0)	**0.246**
Unobvious	43 (43.0)	35 (35.0)	

*Note:* Data are *n* (%). Bold values were no statistically significant difference in the proportion of subjects with ‘significant improvement’ in pore size and skin colour between the topical preparation and IPL, which suggests that the improvements were comparable between the two. The degree of improvement in the above indicators is comparable.

### 3.3 Comparison of Efficacy Within and Between TS and IPL Groups—Objective Measurements

Additionally, dermoscopy was performed to photograph and measure physiology parameters of the partial skin. As shown by Table 4, the values of hydration and elasticity increased significantly after treatment in both groups (*p* < 0.05). For erythema, sebum and melanin, both groups also showed statistical reductions (*p* < 0.05). As for pores, only the IPL group presented a more notable decrease in values after treatment (*p* < 0.05). Similarly, the variations (ΔT_b_ = T_Baseline_—T_1Month_) of the above items’ scores were evaluated to compare the two groups. In comparison to TS group, IPL showed better efficacy in CEA and sebum (*p* < 0.05). However, with the efficacy of moisture, elasticity, pores, and skin sebum, the TS group was comparable to the IPL group (NS).

**TABLE 4:** Dermoscopy measurement scores in the TS‐ and IPL‐treated groups at baseline and 1 month.

Items	TS (*n* = 100)			IPL (*n* = 100)			△T_b_ TS vs. IPL^a^
	Baseline	1 Month	*p*	Baseline	1 Month	*p*	*p*
Hydration, M	1.57 (0.47)	1.76 (0.58)	**0.021**	1.65 (0.58)	1.79 (0.94)	**0.031**	0.891
Elasticity, M	1.65 (0.60)	1.92 (0.48)	**0.004**	1.71 (0.66)	2.04 (0.69)	**0.001**	0.806
Erythema, M	1.09 (0.18)	0.80 (0.12)	**< 0.001**	1.28 (0.14)	0.76 (0.13)	**< 0.001**	**< 0.001**
Pore, M	1.79 (0.35)	1.73 (0.40)	0.065	1.81 (0.37)	1.72 (0.46)	**0.029**	0.663
Sebum, M	1.80 (0.29)	1.61 (0.44)	**0.020**	2.00 (0.33)	1.65 (0.40)	**< 0.001**	0.532
Melanin, M	3.25 (0.75)	3.13 (0.54)	**0.045**	3.15 (0.86)	2.75 (0.63)	**< 0.001**	**0.001**

*Note:* Date (mean (SD)) for hydration, elasticity, erythema, pore, sebum, and melanin were calculated based on skin analysis device (API‐100) measurements, with maximum value = 5. Bold value indicates that the improvement in the corresponding indicators after treatment with the topical serum and IPL were statistically significant.

^a^ △T_b_ TS vs. IPL: compare the variations (ΔT_b_ = T_Baseline_—T_1Month_) of each item between these two groups.

### 3.4 Consideration of Treatment Choice in Subjects With “Obvious Improvement” Erythema in Both Groups

**FIGURE 3:**
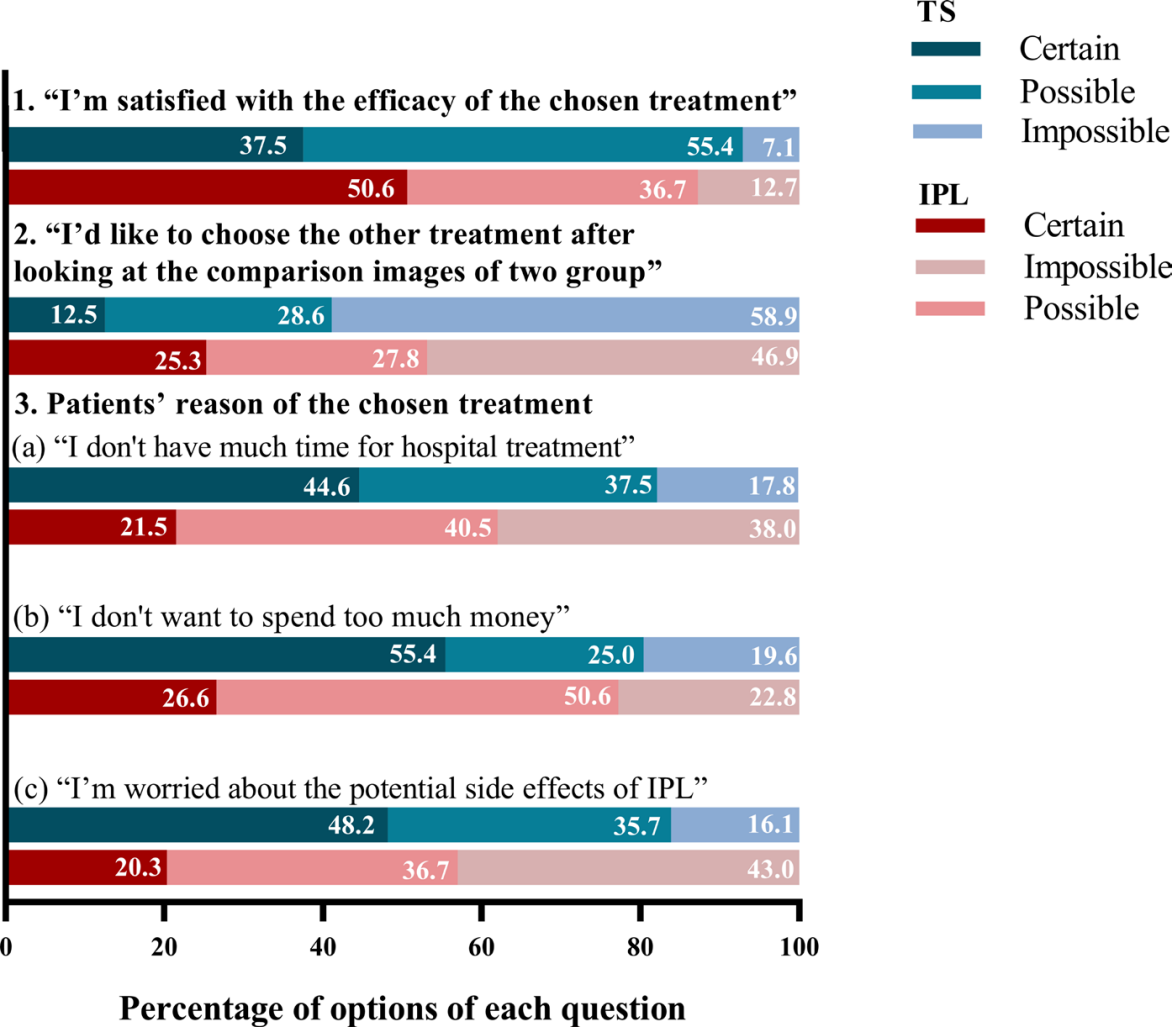
Histogram of the percentage of different choices for both groups.

**FIGURE 4:**
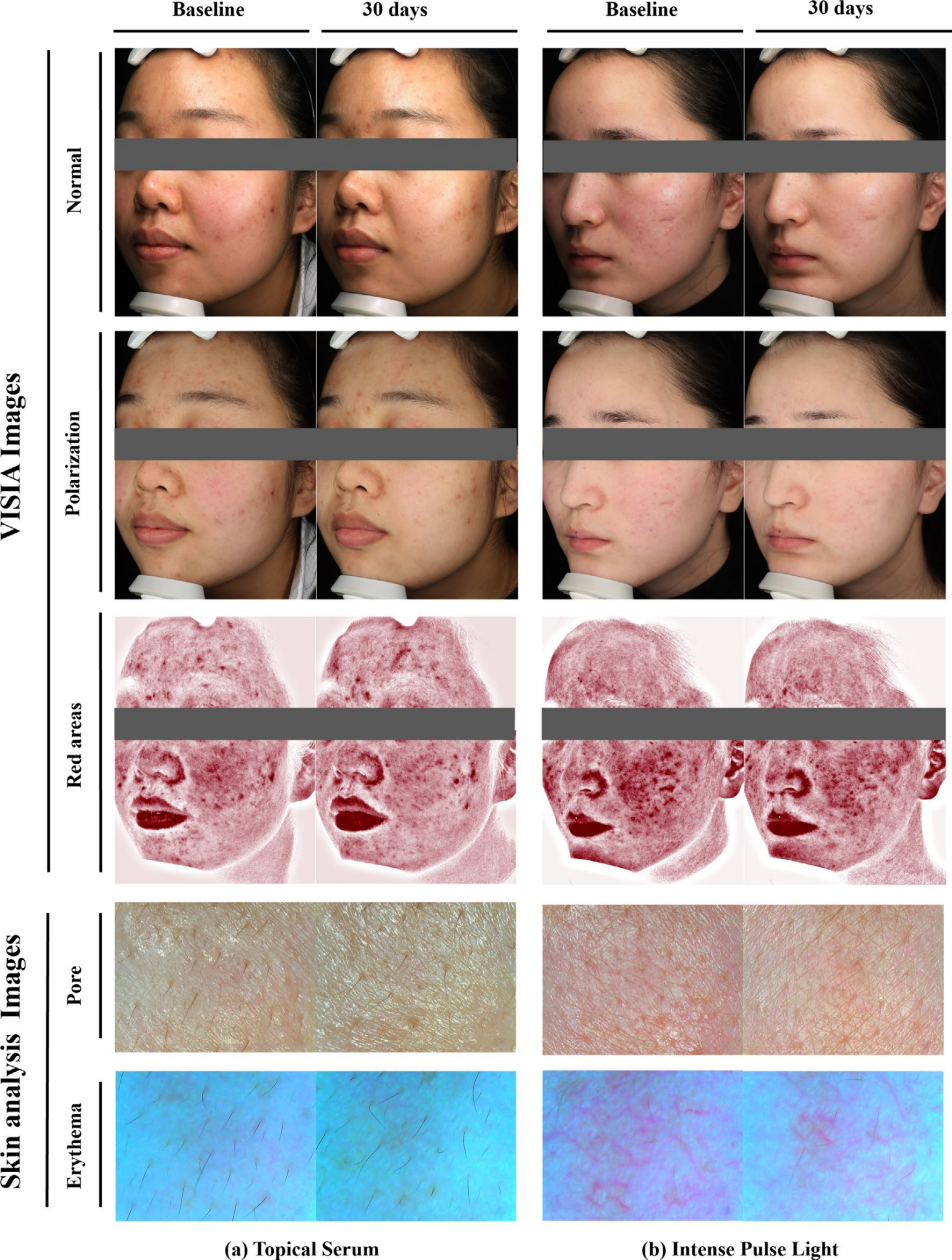
VISIA and skin analysis device of representative clinical example. (a) Before and after 30 days of topical serum application. This is a 23‐year‐old female with a pre‐treatment erythema (CEA = 2) and VAS = 6. After 30 days, CEA = 1 and VAS = 4. With 35% overall improvement, 35% erythema improvement, 30% pore improvement, 10% sebum improvement and 25% skin tone improvement. The skin analysis device showed the skin of the zygomatic area at baseline. After 30 days of treatment, a slight reduction in facial redness can be observed. (b) Before and after 30 days of a single intense pulsed light (IPL) treatment. A 23‐year‐old female, with a pre‐treatment erythema (CEA = 2) and VAS = 7; after 30 days, CEA = 1 and VAS = 4. With 45% overall improvement, 45% erythema improvement, 40% pore improvement, 30% sebum improvement and 40% skin tone improvement. The clearly dilated capillary network was observed by dermoscopy at baseline. After 30 days of treatment, the dilated vessels were significantly reduced, and pores became smaller.

In order to further investigate the subjects' satisfaction with their chosen treatment and the factors influencing their decision‐making, this study further designed the questionnaire, in which subjects with “Obvious improvement” erythema participated (56 in TS group, 79 in IPL group). As shown in Table 5 and Figure 3, the satisfaction with treatment was similar in both groups of subjects (NS). When they were further asked “If I had another chance, I'd like to choose the other treatment,” after being shown pre‐ and post‐ images of effective and ineffective treatment, 46.9% of subjects in the IPL group wanted to choose the same treatment, in comparison to 58.9% in the TS group. However, there was no statistically significant difference between the two groups' choices (NS). As we projected, treatment cost (time and price) and potential side effects of IPL were considerations for most of the subjects who chose TS. By contrast, subjects in the IPL group tended to trust friends/social platforms and were more willing to pay for treatment. The representative clinical images of two patients treated by TS and IPL are suggested in Figure 4.

**TABLE 5:** Subjects' attitudes toward TS and IPL.

	TS (*n* = 56)	IPL (*n* = 79)	*p*
“I'm satisfied with the efficacy of the chosen treatment”			
Certain	21 (37.5)	40 (50.6)	0.161
Possible	31 (55.4)	29 (36.7)	
Impossible	4 (7.1)	10 (12.7)	
“I'd like to choose the other treatment after looking at the comparison images of two group”			
Certain	7 (12.5)	20 (25.3)	0.103
Possible	16 (28.6)	22 (27.8)	
Impossible	33 (58.9)	37 (46.9)	
Subjects' reason of the chosen treatment			
“I believe in the effect of IPL from friends/social platforms”			
Certain	16 (28.6)	45 (57.0)	
Possible	27 (48.2)	19 (24.0)	
Impossible	13 (23.2)	15 (19.0)	
“I'm worried about the potential side effects of IPL”			
Certain	27 (48.2)	16 (20.3)	
Possible	20 (35.7)	29 (36.7)	
Impossible	9 (16.1)	34 (43.0)	
“I don't have much time for hospital treatment”			
Certain	25 (44.6)	17 (21.5)	
Possible	21 (37.5)	32 (40.5)	
Impossible	10 (17.8)	30 (38.0)	
“I don't want to spend too much money”			
Certain	31 (55.4)	21 (26.6)	
Possible	14 (25.0)	40 (50.6)	
Impossible	11 (19.6)	18 (22.8)	
“I have no faith in the ability of topical serum to improve facial condition”			
Certain	8 (14.3)	36 (45.6)	
Possible	17 (30.4)	38 (48.1)	
Impossible	31 (55.3)	5 (6.3)	

*Note:* Data are *n* (%).

## 4 Discussion

Consistently, many efforts have been made to treat rosacea. Oral doxycycline (40 mg per day) has been approved by the FDA for treating rosacea because it has fewer adverse effects. However, it is much more effective in eliminating papulopustular lesions [17] and not as effective for flushing and mild erythema. Brimonidine tartrate gel (α_2_‐adrenergic agonist receptor) was also approved by the FDA in 2013 for the treatment of persistent erythema (moderate‐to‐severe) and flushing in adult rosacea [18]. However, as reported by studies, patients have experienced intense rebound erythema during use or after discontinuation [19, 20]. According to available studies, the therapeutic effects of both oral and topical medications are mostly unsatisfactory. For patients who are plagued by mild erythema and want to further reduce symptoms, IPL seems to be a good choice, which undoubtedly resolves some of the fixed erythema, capillary dilatation, and proliferation [21]. However, it inevitably leads to perturbation of the skin barrier and intolerance in some patients [22]. Additionally, significant time and economic costs are associated with IPL [12], and evidence‐based medical support for IPL of rosacea with mild persistent erythema is insufficient [23, 24].

Simple methods for reducing mild erythema in rosacea continue to be explored. Among them, topical preparations with anti‐inflammatory, skin‐hydrating properties, and reduction of fixed erythema are hot topics in research [1, 25]. However, there are deficient comparative studies on the similarities and differences between these topical preparations and intense pulsed light, in efficacy, safety, and patient satisfaction. Rosacea patients with mild erythema who are suitable for IPL can choose their treatments independently (topical preparations vs. IPL). Accordingly, a moisturizing serum (Redness Neutralizing Serum, NeoStrata Company Inc., Princeton, NJ) based on PHA (5% gluconolactone and 1% lactobionic acid) was chosen for observation in this study. Gluconolactone is recognized to improve skin barrier function by reducing trans‐epidermal water loss (TEWL) and exerting a positive effect on enlarged pores [26]. Lactobionic acid can be chelating agent with matrix metalloproteinase (such as MMP9) [27], to reduce skin laxity and telangiectasia. It also has good moisturizing properties to improve dryness, redness, and itchy skin conditions [28]. To the extent of our knowledge, this is the first inaugural study to examine the enhanced efficacy of this Redness Neutralizing Serum with intense pulsed light on rosacea.

All investigator assessments have shown improvement after treatment in both groups (*p* < 0.05). As revealed by treatment variations between the two groups (investigator assessment and measurement), IPL was more effective in VAS, CEA, and sebum (*p* < 0.05). This might be explained by the broad spectral characteristics of IPL, to seal blood vessels, reduce inflammation, improve pigmentation, increase collagen (type I and III), align elastic fibers, and inhibit sebaceous glands activity at the same time [21, 29, 30]. The combined effect resulted in a significant overall facial improvement. However, for TS, the mitigation of erythema may be accomplished through the alleviation of inflammation, with a limited capacity to curtail capillary dilatation [27]. No significant differences were found between the two groups in terms of pores and skin tone with positive results for each subject. This may be because rosacea inherently has high facial skin fineness and smaller pores, resulting in limited improvement. The results of dermoscopy measurements were similar to those of the subjective assessment. Additional measurements of skin hydration and elasticity showed significant improvement in both treatment groups (*p* < 0.05) (Table 4, Figure 4), which affirms the moisturizing ability of TS and that a single IPL treatment will not have a negative effect on skin hydration.

Through analysis of the before and after photographs of the subjects, it can be observed that those with erythema combined with melasma and capillary dilatation, who were considered to reduce erythema, evened out skin tone and reduced capillary dilation through IPL treatment. However, not all patients harboring these conditions selected IPL for treatment. In order to explore the underlying reasons for such occurrence, a questionnaire was designed. Only a few subjects in both groups were dissatisfied with their choice of treatment (7.1% in TS vs. 12.7% in IPL). This is similar to previous study that meaningful choices can enhance patients' sense of autonomy and self‐efficacy [31]. When reviewing the comparison images, this study found that some subjects wavered in their choice of treatment. Fewer subjects choosing “Certain” in TS group was less compared to the IPL group (12.5% vs. 25.3%), who felt that TS did improve redness. However, IPL had definitely shown better results in further resolving facial problems (friends/social platforms/images), and they these subgroup subjects were willing to pay more (time and cost) for hospital treatment. By contrast, participants who chose “Possible” reported being hesitant about finances and side effects of IPL, with redness reduction being their main concern. In IPL group, 53.1% of subjects chose “Certain” or “Possible”. After looking at the images, their perception of TS changed and appreciated the improvement of redness with TS, indicating that it had achieved their treatment goal. Subjects who chose “Possible” were more concerned about their compliance to continuously use it.

It is crucial to acknowledge the limitations of our study. This retrospective study was constrained by objective factors and only focused on documenting changes in objective skin indicators but did not inquire about changes in other signs and symptoms, such as burning, pain, itching, and flushing. Meanwhile, there might have been a recollection bias among the subjects, which could have influenced the accuracy of their responses. In future trials, we will prospectively observe the improvement of subjective symptoms and the long‐term use of this serum to further understand its efficacy.

In conclusion, intense pulsed light reduces erythema and is superior in improving the overall facial appearance. For patients who only want to resolve redness and factor in cost and possible side effects, IPL is not advantageous. Serums that reduce redness, improve skin hydration, and strengthen skin barrier are low‐cost in terms of time spent on daily skincare, simple to operate, and relatively gentle. The further development of such redness‐reducing preparations is vital and is an alternative for erythematous patients who are unable to undergo IPL.

## Author Contributions

All authors contributed to the study conception and design. Yiran Peng: Analyzed the data, visualized the results, and wrote the manuscript. Mengping Mao: Collected the data and revised the manuscript. Dan Jian: Designed the study, suggested the conception of the work, and revised the manuscript. Other co‐authors: Supervised the clinical work, contributed in the revision of all the data, and shared in writing the whole paper.

## Ethics Statement

This research study was conducted retrospectively from data obtained for clinical purposes and was performed in line with the principles of the Declaration of Helsinki. Approval was granted by the Ethics Committee of the Institutional Research Ethics Board of the Xiangya Hospital, Changsha, China (ID: 202305099) and registered in the Chinese Clinical Trial Registry (ChiCTR2400087665).

## Consent

Exemptions have been granted from the requirement to obtain written informed consent.

## Conflicts of Interest

The authors declare no conflicts of interest.

## Data Availability

The authors have nothing to report.
